# The Hospital. Nursing Section

**Published:** 1902-12-27

**Authors:** 


### The Hospital.

### Nursing Section.

Contributions for this Section of "THE HOSPITAL" should be addressed to the EDITOR, "THE HOSPITAL" NURSING SECTION, 28 & 29 Southampton Street, Strand, London, W.C.

No. 848.—VOL. XXXIII. *SATURDAY, DECEMBER* 27, 1902.

### Notes on News from the Nursing World.

### OUR CHRISTMAS DISTRIBUTION OF CLOTHING.

THE garments sent to us for distribution at Christmas were made made up into parcels last week and forwarded to the following hospitals:—Belgrave Hospital for Children, 22 articles; British Lying-in, 28; City of London Hospital for Diseases of the Chest, 12; East London Hospital for Children, 30; East End Mothers' Home, 22; Great Northern Central Hospital, 18; Guy's Hospital, 15; King's College Hospital, 15; Metropolitan Hospital, 13; North London Hospital for Consumption, 11; Paddington Green Children's Hospital, 16; St. George's Hospital, 19; and St. Mary's Hospital, Paddington, 10.

### THE KING'S INVESTITURE.

LAST Thursday the King held an Investiture at Buckingham Palace, and bestowed the decoration of the Royal Red Cross on the following Nursing Sisters:—Miss Sophia Margaret Paterson, Miss Katherine Blanche Brereton, Miss Emily Margaret Whiteman, of the Army Nursing Service Reserve; Miss Joan Charleson, Mrs. Sophie Lees, Mrs. Harriet Maude Campbell-Ross, and Miss Caroline Helen Kerr. The other recipients of the Order were Mrs. Margaret Scott Fripp, Miss A. J. Weighell, superintendent of the Countess Roberts's Hospital and Nurses' Home in India, and Miss Ker Dunlop. The services rendered by Miss Dunlop in connection with the care of sick and wounded soldiers during the South African war consisted in the establishment and maintenance of a home in Perthshire for their reception.

### "THE PERSONAL CHARGE OF QUEEN ALEXANDRA."

IN her communication to the women of England and Wales inviting them to enable the executive committee of the Women's Memorial to Queen Victoria "to put the coping-stone to the edifice of which Queen Victoria and the women of the Empire laid the foundation in 1887," Lady Londonderry, confirming our statement of last week that the result of the memorial appeal will be handed to the King and Queen early next year, reminds the public that the Queen's Nurses "have now become the personal charge of Queen Alexandra, whose first public act, since the accession of the King, was to welcome them from all over the kingdom to Marlborough House." In other words, the Queen's Nurses remain, in the most literal sense, the Queen's Nurses; and those who respond to the final appeal of the committee to offer to the King and Queen as worthy a gift in memory of Queen Victoria and for the help of the suffering poor as the Jubilee gift of 1887, will have the satisfaction of feeling that they are not merely doing honour to the beloved and illustrious dead, but also to the beloved and illustrious living.

### THE SCOTTISH BRANCH OF QUEEN VICTORIA'S JUBILEE INSTITUTE.

THERE is one unsatisfactory feature in the report which was submitted at the fourteenth annual meeting in Edinburgh of the Scottish branch of Queen Victoria's Jubilee Institute for Nurses. The chairman, Sheriff Guthrie, K.C., in moving its adoption, pointed out that 31 nurses had completed their training during the year; that 11 new branches had been started by local committees; and that the total number of Queen's nurses in Scotland is now 224. But, unfortunately, the expenditure exceeded the receipts by no less a sum than £830. The Council had, therefore, as the report says, to draw upon their "small amount of available invested funds." This is the more serious because for the last five years the average withdrawals from invested funds to meet over-expenditure have been nearly £650. Under these circumstances, the Council rightly regard "with anxiety the insufficiency of income to cover the increasing and necessary demands made upon their resources from year to year." We note that they believe that the work is increasingly appreciated, and cite in support of this view the fact that the number of donations in Edinburgh, either by patients themselves, or by friends on their behalf, has grown larger, and has been given in sums which vary from 1s. to £4. But the very idea of the income continuing to be inadequate should not be tolerated, and all the friends of the branch in Scotland should strenuously address themselves at once to the task of putting it on a sound financial basis.

### THE REPORT ON WORKHOUSE NURSING.

WE understand that the Executive Committee of the Workhouse Infirmary Nursing Association have met in order to consider the report of the Departmental Committee on Workhouse Nursing, and have appointed a special sub-committee to further go into the various points.

### THE "QUALIFIED" NURSE IN IRELAND.

AT a time when the Local Government Board in England are advised by a committee of their own nomination to create a new order of "qualified" nurses the Local Government Board in Ireland are refusing to allow the Tuam Board of Guardians half the salary of a nurse for the workhouse because, in contravention of their instructions, the Guardians appointed what they called a qualified instead of a trained nurse. That is to say, the nurse selected possessed certain qualifications which in the estimation of the Guardians was sufficient, but had not been trained in accordance with the regulations insisted upon by the Local Government Board. The situation is not without humour, but it is a matter for satisfaction that the

Irish Local Government Board are determined to maintain the standard of nursing which they have laid down.

### THE SALARY QUESTION IN THE CROWN COLONIES.

It will be remembered that in our issue of November 15 a correspondent cited extracts from a document issued under the auspices of the Colonial Office foreshadowing a reduction in the salaries of nurses employed in the civil hospitals in the Far East, particularly Hong Kong. It remains to be seen whether her fears will be realised. In the meantime, as we now understand, the view of the Colonial Nursing Association, whose communication to the Colonial Office was limited to an inquiry respecting the nature of the new terms, is that they are considerably better than the old. This opinion appears to be chiefly based on the ground that as nurses are now to be paid in sterling, besides knowing exactly what their income will be, they will pay for their food in dollars, and will therefore profit by the rate of exchange. By the former arrangement half the salary was computed at one rate of exchange and half at another.

### NURSING MINERS IN WEST AFRICA.

At the annual meeting of the shareholders of the Taquah and Abosco mine, it was stated that there had been no deaths among the miners for 18 months, although there had been many cases of sickness. The good recoveries made were attributed to the fact that the European Hospital at Abosco, which is fully equipped, is at the service of the men. At this hospital, which is in charge of a medical man, the nursing is done by Miss E. M. Swire, a trained nurse, who has enjoyed previous experience in West Africa. A Cornish workman just returned from that unhealthy coast, enthusiastically referred to the hospital as "the best in West Africa," and to Miss Swire as "the most attentive and considerate of nurses."

### DARLINGTON FEVER HOSPITAL.

The appointment of Miss Beatrice James to the post of matron of Darlington Fever Hospital was not decided upon without difficulty. There were no less than 64 applications, many of them from very suitable persons, and the final selection was not made until last week. Miss James, who is a daughter of the Medical Officer of Health for Prescot, has had the advantage of varied experience in the provinces.

### THE NURSES OF WAKEFIELD INFIRMARY.

A very interesting ceremony took place in the Nurses' Lecture Hall of the Wakefield Infirmary on Wednesday, December 17th, when silver medals were presented to Sister Margaret Quinn and Nurse Matilda Hainsworth for having passed with credit the recent examination in midwifery. Four nurses were examined and three passed, two having gained the silver medals offered by the Chairman of the Board of Guardians and the Chairman of the Infirmary Committee. Mrs. Dixon, one of the lady guardians, made the presentation, and speeches were delivered by Miss Lightowler, Superintendent; Mr. C. Holliday, chairman of the Committee; Mr. W. Moorhouse, chairman of the Board; Colonel Maddison; and Mr. Stonehouse. On the same occasion Nurse Florence Birch was presented with her certificate on passing the examination at the completion of her three years' training. Most of the guardians and the nurses were present, and afternoon tea was served.

### CHESTER DISTRICT ASSOCIATION.

While the Chester District Nursing Association must be congratulated upon the results of the bazaar, which yielded a total of nearly £200, the citizens, it may be hoped, will not rely upon special efforts of this kind to keep the organisation in funds. At the opening of the bazaar it was explained that, though the five nurses had paid some 16,000 visits up to the middle of December and their work finds increasing appreciation, sufficient money is difficult to obtain. Last year, for instance, the subscriptions only amounted to £350, as against the £600 absolutely needed, and, if it had not been for a legacy, the Association could not have paid its way. The bazaar will doubtless square matters for 1902, but it cannot be too strongly impressed upon the public that if they wish five district nurses to be employed in Chester, it is their duty to free the managers from anxiety about the wherewithal by affording sufficient regular assistance.

### ACTON COTTAGE HOSPITAL.

Our attention has been called to the fact that the matron of the Acton Cottage Hospital recommended one of her probationers, aged 23, for the post of district nurse, and that the Council, in the absence of other candidates for the appointment, selected her. Dr. Rudd protested against the choice on the ground that a probationer who has been "trained" for one year in a hospital with seven beds is not fully qualified. No one can know this better than the matron, who was herself trained at the Royal Hospital for Sick Children, Edinburgh, and the London Temperance Hospital; and no attempt was made either by her, or by the members of the Council who supported the appointment of the probationer, to maintain that the latter had received the recognised training.

### HUDDERSFIELD GUARDIANS AND DISTRICT NURSES.

An appeal has been made to the Huddersfield Board of Guardians to assist the funds of the Huddersfield and District Victoria Nursing Association. The force of the plea can hardly be denied, and it is surprising that it has not been successfully urged before. During last year the district nurses attended no fewer than 120 operations many of the patients being persons receiving outdoor relief. It is obvious that but for the nurses these cases must have been taken either to the infirmary, or to the workhouse. In the former event the ratepayers would not, it might be contended, have directly suffered in pocket. But in the latter they certainly would, and the Huddersfield people doubtless desire their representatives to be fired by the example of the Leeds Board which in similar circumstances renders substantial help.

### THE DUAL AUTHORITY AT BELFAST.

It is significant that at the annual meeting of the Belfast Nurses' Home and Training School, the prospect of the dual authority in management which has hitherto been in force is coming to an end, was hailed with satisfaction. The Royal Victoria

Hospital will be transferred in the spring to its new quarters, and thenceforward will be able to accommodate its own nursing staff. There have been the most pleasant and cordial relations between the home and the hospital throughout. Yet, there is no doubt that both institutions will gain by the impending change. It is always an immense advantage for a hospital to have its own staff of nurses under its own roof, and on the other hand there is ample scope in Belfast for the private nurses who will in future have exclusive possession of the home. Thirty-one years ago, before the home was established, the Victoria Hospital nurses were housed in the basement, there was no trained superintendent either by night or by day, no lectures, no examinations, no annual holiday, no proper time of off duty. The nurses had to cook and eat their food in the ward kitchen, when they could snatch a few minutes to do so; they had also to carry the coals from the hospital yard to the top of the building, wash the ward-floors and steps of the hospital stairs, and do their own laundry work. With the advent of the Home the situation changed. A four years' system of training was introduced, the standard of efficiency was raised, the nurses were comfortably housed and boarded, menial duties were abolished, off-duty time was arranged, holidays were given, and, in fact, the home has, from the outset, had all the characteristics of an excellent training school. Since it was founded, at least 300 nurses have been trained there, and have gone out to positions of trust, while the supply of private nurses has never been equal to the demand.

### THE SALARIES OF SUTHERLAND NURSES.

It is an excellent thing to have a good balance at the bank, but the first duty of a District Nursing Association is to pay its nurses properly. There are thirteen nurses attached to the Sutherland Nursing Association who receive £328 per annum between them. In any case this is certainly too little, and with a balance in hand of upwards of £700, it cannot be pleaded that the scanty salaries are due to lack of means. As a subscriber to the Association says: "To think of a woman undergoing a course of hard training and qualifying for a nurse, and starting on £20 per annum, rising by £2 to £30 and stopping there, is a fact the originators of the organisation ought to seriously consider."

### PRIVATE AND DISTRICT NURSING AT MANCHESTER.

The feature of the report of the Manchester and Salford Sick Poor and Private Nursing Institution, which was adopted at the annual meeting the other day, was the serious falling off in the income. The financial statement showed that the institution is £200 worse off than it was at the same period in 1901. We are sorry that an organisation which has been in existence for more than a quarter of a century should be in this position. But we fear that it is partly due to the combination of two branches which it is better to keep separate. It was stated at the meeting that the private nursing department had been able to hand over to the general fund towards the support of the nurses who work among the sick poor the sum of £230. This is a bad principle. Instead of the earnings of private nurses being utilised for the purpose of defraying the maintenance of nurses attending the sick poor, the money required for the latter should be collected from the public. Manchester should be in the van of the nursing movement, not in the rear.

### A NEW NURSING ASSOCIATION FOR NORTHAMPTONSHIRE.

It has been decided to form a County Nursing Association for Northamptonshire. At the drawing-room meeting in Milton Hall, several speakers, including Miss Amy Hughes and Miss Saunders, lady superintendent of the Peterborough Nursing Association, advocated its establishment on the lines of the associations already affiliated to Queen Victoria's Jubilee Institute. Miss Hughes has lately set forth in our columns the manner in which these Associations are worked, but though there is force in her contention that a half-trained nurse is better than none at all, we regret that the promoters of the new movement in Northamptonshire have not been influenced by the success of Lady Knightley and her friends in poor districts adjoining Daventry—each of which employs a nurse who has had three years' training in one of the best hospitals—to aim high at the outset.

### A GOOD BEGINNING AT WATERFORD.

It is pleasing to record the successful formation of the Waterford District Nursing Association. At the public meeting held in support of the movement, there were representatives of various religious bodies on the platform, the Roman Catholic Bishop of Waterford being in the chair. The origin of the movement was the engagement in December 1900 of one nurse under private auspices. By the spring of this year, however, her work had so increased that in June a second nurse was engaged, and as she also speedily became fully occupied, it was decided to start a regular organisation.

### SHORT ITEMS.

Until the Privy Council has sanctioned the appointment of a secretary to the Midwives' Central Board, Mr. Heywood Johnstone, M.P., is acting as secretary. The chief duty of the permanent official will be the custody, under the direction of the Board, of the roll of midwives directed to be prepared under the Act.—Messrs. Archibald, Daniel, and Peter Coats, of Paisley, have given £1,000 to the endowment of the Ramsey Nursing Home, which was opened by Princess Henry of Battenberg to celebrate the Diamond Jubilee of Queen Victoria.—A nurse named Glendenning, residing in Belfast, has instituted an action claiming damages £1,500 for breach of promise of marriage against Dr. John Byers. The defence is a denial of the promise alleged.—Miss Amy Burgess, the new superintendent nurse at the Cuckfield Union, was, we are informed, nurse, and not sister, at Woolwich Infirmary, Plumstead.—An entertainment was given to the in-patients of the Cancer Hospital, at Brompton, on Thursday last week, by the Rev. J. L. Geffen and party. The programme contained songs, recitations, conjuring, ventriloquism, &c., and concluded with a sketch entitled "Between the Posts."—The name of the nurse whom Dr. Murphy, of Naas, declined to employ is not Short, but Welsh.

### Lectures to Nurses on Medical Nursing.

By Eveline A. Cargill, M.D.Brux., L.R.C.P.&S.Ed.

### LECTURE XIII.—INFECTIOUS FEVERS.

(*Continued from page* 130.)

*Typhoid or Enteric Fever* differs from other infectious fevers in the gradual and indefinite way in which the symptoms appear, and in the fact that it is infectious only through the evacuations of the patient, so that a typhoid case may be safely nursed in hospital or private house without isolation, provided special care is exercised over all evacuations of the patient. Enteric fever attacks all ages and both sexes, but especially young adults, it lasts three weeks, and is liable to relapses, and its distinctive features are an eruption of pink spots and diarrhœa with characteristic stools. The *incubation* period is variable, about 14 days. The *invasion* is gradual, the patient has headache and feels ill, but can often struggle on for a week or more before going to bed. The temperature rises all this time, and is now about 103° at night and 101° in morning. Gradually the patient assumes the characteristic "typhoid" aspect, is dull and apathetic, with bright eyes, pale face with flushed cheeks and dark lips, tongue dry with white fur at sides and red in the middle. There is severe headache. At the end of the first week the rash appears, a few pink spots on chest and abdomen, and lasts till the end of the third week. The abdomen becomes distended and tender, and there is diarrhœa; three or four motions daily of a distinctive character, like "pea-soup," liquid, yellow, and very offensive. Hæmorrhage from the bowels is fairly usual, and may be severe. During the second week the temperature remains high, 103° or 104° at night and 101° in morning; at the end of the week it begins to come down till it reaches the normal, and convalescence sets in. This is a mild attack—the whole illness may be much more severe, there may be delirium or coma and extreme prostration, or abdominal complications; peritonitis from perforation of the bowel is an ever-present dread in typhoid fever, and may occur in mild cases from apparently slight causes. If perforation takes place there is first collapse, and then peritonitis, which is nearly always fatal. Relapses are not uncommon even after the patient is apparently convalescent, and then the whole illness may be repeated. There is no illness which presents so many variations as enteric fever or so many complications, and there is no illness where careful, intelligent nursing is so essential. The great point to remember is the danger of perforation, therefore the patient must be kept absolutely at rest, no exertion whatever being permitted. He must be kept clean by sponging all over daily with tepid water, and all soiled linen and bed-clothes removed immediately and placed in a covered pail containing perchloride solution (1 in 500), the pail being brought to the bedside and then immediately removed from the room. Diet is very strict, generally milk only; all cups, etc., must be kept for the patient's use only. If diarrhœa is excessive it may have to be treated with enemata, and these must be very carefully given without disturbing the patient. Before giving him the bed-pan, a little disinfectant solution (perchloride 1 in 100) should be put in it; after using, more disinfectant added and the vessel thoroughly cleaned. All bed-pans should have covers, and be removed (covered) from the room directly after using. If a motion is kept for inspection, it must be in a covered vessel with a cloth (which can be afterwards burnt), wrung out of perchloride solution (1 in 100) under the lid. Before throwing away more strong disinfectant must be added to the stool. A basin of carbolic solution (1 in 20) by the bedside is for the nurse to dip her hands each time after touching the patient; the thermometer should be dipped after using.

*Scarlet fever* has a short incubation period, two to seven days; the invasion is sudden, with rigor, vomiting, headache, sore throat, high temperature (103° or 104°), and a very rapid pulse (120 to 160). The rash appears on the second day, first on chest and neck, but spreads rapidly over the body. It consists of minute raised spots of a brilliant red; the face is flushed, but has no rash. The rash lasts five days or more, and is followed by "peeling," the outer skin being shed in scales or large flakes, but there may be only a little roughness. The complications are often serious; ulcerated sore throat, ear troubles, rheumatism, and kidney disease are frequent; hence the need for careful protection against cold or draught, especially during convalescence. Strict isolation is necessary, even for mild cases, until peeling is finished, and this takes six weeks at least. Warm baths night and morning, with carbolic soap, help to remove the scales, and the skin may be smeared with oil to prevent the scales floating away, as infection is conveyed by means of the skin. As the contagion of scarlet fever is very active, disinfection of patient and room, etc., must be very thoroughly carried out.

*Measles* has an incubation period of ten days, and a fairly sudden invasion, with a rise of temperature, vomiting or convulsions, watery eyes and nose, and a slight cough. The temperature falls after the first day, and rises again with the appearance of the rash on the fourth day to 103° or higher. The rash comes out first on the face, forehead, round the hair, and behind the ears, and then spreads to neck, trunk and limbs. It consists of pink spots, which run together to form irregular patches of a dark purply shade. It is most marked on the face, to which it gives a peculiar blotchy, swollen appearance. It fades in one to three days in the same order in which it came out, and is followed by slight peeling in fine scales. In itself measles is generally a slight illness, but it is so often complicated by affections of the lungs and throat, inflammation of eyes and ears, and leaves behind it so many chronic diseases, that it is more to be dreaded than many more serious illnesses. The treatment and isolation is the same as for other infectious fevers; the patient should stay in bed as long as the rash is out, and special care being taken to avoid draughts and cold, and isolation should be maintained for ten days after the rash has faded.

*German Measles* is a distinct disease though resembling ordinary measles. The rash is pink, and does not run together, the glands in the neck are swollen, the catarrh is slight, as is the whole illness, and there are practically no complications.

*Diphtheria* has a short incubation period of two to six days, and the invasion is gradual, with headache, loss of appetite, and soon sore throat. The tonsils and uvula are seen to be red and swollen, and soon creamy white patches appear on them, which unite to form a "false membrane," the glands of the neck are enlarged. The temperature does not run very high, 102° or lower; the pulse is rapid and feeble, and the patient rapidly loses strength. Diphtheria of the larynx was described in the lecture on respiratory diseases, but the patient may die of general poisoning without the larynx being affected at all, or he may die suddenly from heart failure. Diphtheria is often followed by paralysis, which shows first in the soft palate giving a nasal tone to the voice, and is often limited to this, but it may affec all

### LECTURES TO NURSES ON MEDICAL NURSING.—*Continued.*

the muscles of the body and cause death when respiration is interfered with.

*Small-pox* is virulently contagious, not only by contact with the patient but by breathing the same air with him, and is carried by clothes, bedding, etc. A small-pox patient is infectious both before the rash comes out and after death as well as during the disease itself. The incubation is 12 days, the invasion sudden, with rigors, pain in the back, headache, high temperature, and the rash appears on the third day as little raised red spots on forehead and face, later on trunk and arms and legs. The spots become purulent with intense surrounding inflammation, and then gradually dry up and leave a scar. The fever runs very high and the suffering is great; there are many and serious complications, and the mortality is high. The treatment is the same as for other fevers, and isolation must be very strict, but small-pox differs from other fevers in being almost entirely preventable by efficient vaccination, and even if contracted after vaccination is then so modified as to be a comparatively mild disease.

### Christmas in North Germany.

BY AN ENGLISH NURSE.

### In the District.

Germany, as all the world knows, is the land of Christmas trees and birthday fêtes, and my own experience as a probationer in a German hospital may be interesting to English nurses. During the last fortnight before Christmas the district nurses are always at work till about 12 p.m., packing up parcels and dividing the presents which so many people kindly send for the purpose. These are given away a few days before Christmas, to the poorest or most deserving patients. Owing to the kindness of a district nurse I was allowed to help at the distribution.

When at length the happy evening arrived, we dressed in our best black cashmere dresses (our working dress is blue cotton), clean caps, and aprons, and off we started. It was a wild night and very dark. We entered a large hall, and, coming from the darkness outside, it seemed like fairyland. It was a very lofty wide room, at one end a small platform with a harmonium, and on each side two huge Christmas trees lighted by numerous candles. Two large silver stars at the top looked very pretty, glittering and shining in the candle light, recalling to one the Star of Bethlehem. The walls were hung with dark-green garlands of fir-tree boughs. Extending down the room were four long tables on trestles. These were covered with snow-white cloths; two lighted Christmas trees stood on each table, and at the other end of the room was another tree which towered above all the others. All the trees were lighted with numerous candles, decorated with silver stars, pretty texts, and very pretty paper chains, etc., made by the children in the district in times of convalescence. The tables accommodated in all 320 people, but 400 were present.

Each visitor received a long loaf of white bread with currants or sultanas—a delicacy for poor Germans, who can only afford to buy brown bread—a bag of pfeffernüsse, reminding me of English gingerbread, and two or more useful articles of clothing. Nor had the children been forgotten, as I saw many dolls, picture-books, whistles, etc. On each place was a white card with the name attached, so that there should be no mistake, and all the places were arranged as far as possible according to the streets the people lived in.

As the clock struck seven the people were allowed to come in—a stream of poor women and children. There were very few men—they were only permitted to be present if no other member of the family was available.

One seldom, even in the worst slums of Hamburg, sees such terrible poverty in Germany as in England. The women were all in their best black clothes—the German poor and middle-class people always wear black on special occasions—looked so neat and nice. None, if possible, would like to miss being present, one of the attractions being the Christmas trees, without which no German, rich or poor, high or low, would feel that Christmas had been properly kept. Many of the mothers, if possessing a number of children, came armed with washing-baskets and big bags, to carry away their gifts.

When all was quiet one of us played a Christmas hymn, the chief clergyman gave a short, but suitable address, after which we sang some carols, the people joining in heartily, and then the distribution began. Later another hymn and a carol were sung, the blessing said, the women shook hands with us—seeming very pleased with their things—curtseyed, and went quietly away.

### In the Hospital.

A few days afterwards it was Christmas Eve. From early morn the ward nurses were busy decorating their trees, we downstairs (I was then only a new pro., I was still in the kitchen) having been given a day off cooking, were allowed to our great delight to decorate the nurses' tree. What a large tree it was; we had to climb on our highest steps to reach the top. We decorated it with silver and gold stuff, glittering balls, artificial snow, and numerous candles. At five we went to the Christmas Eve service at church, next to the wards and lighted the patients' trees, and stood round singing a carol, the matron reading prayers, where the patients could bear. Then we had our supper, and at about nine filed to the committee-room—the matron was already there—and stood round "our" tree, according to rank. It was a magnificent tree, all lighted up, the table covered with a white cloth; the dark green of the fir tree, with the figures of the matron and nurses, with their hair parted smoothly on each side, their quaint white caps and black cashmere dresses, made quite a picture. According to custom on a special festival, we wore no aprons. Oranges, apples, and nuts, very tastefully arranged on the green boughs, were distributed according to our position. The head nurses received six oranges, twelve apples, pfeffernüsse, nuts, etc., other nurses four oranges, etc, and I, as youngest pro., two oranges, four apples, etc. In the centre of each place was a plate of sweetmeats, chocolates, marzipan, also decorated according to position. When we were all assembled we sang carols; then our tongues were loosened and Babel began. Such a hubbub of German! How happy they all were, and so was I, for they were all very kind indeed to me, as the only English nurse. The presents were very nice; the nurses can choose what they wish from the committee, the pros. up to the value of 5s., increasing to 20s. for the sisters. After a time some of us played on the piano—a rather rare occurrence—and sang, but of course only sacred or classical music, as the Red Cross nurses in Mecklenburg-Schwerin are as strict as the deaconesses. At about 11 p.m. we all went to bed, tired and happy.

### The Report of the Departmental Committee on Workhouse Nursing.

(*Continued from page* 168.)

How it Concerns the Public.

We have so far considered the probable effect of the recommendations upon the sick in the workhouses, upon the administration, and upon the nurses and probationers engaged in poor-law work. We have shown that in regard to each of these points there is much reason for believing that their effect will be good, namely that, putting aside for the moment all question of those large infirmaries in which the nursing is already established on a proper basis, the sick in the workhouses will be better nursed than they are at present, that many administrative difficulties will be smoothed away, and that finally the nurses and probationers will have a more comfortable time than they have now. Thus, so far as concerns those aspects of the question which the departmental committee set itself to solve, the result may be described as satisfactory. Far otherwise, however, is it when one considers the probable effects of the recommendations upon the general body of nurses who have to earn a living by the exercise of their art, and upon the general mass of the public who when sick have to trust themselves to such nurses as they can find. To both nurses and the public the effect of the recommendations of the committee must e disastrous, tending as they do to officially re-establish the "one year" standard of training from which the whole profession has for so long been endeavouring to raise itself, and to flood the market with "nurses" who, although only accepted by the poor-law authorities as fitted to act under supervision, will without a shadow of a doubt be regarded by the public as "trained," "certificated," and "qualified." These "one-year trained" women will be able to say with perfect truth that they have been trained in training schools recognised by the Local Government Board, that they possess certificates granted under the sanction of the same august body, and that the title of "qualified nurse," has been specially granted to them. How is the agitated public, running about in search of nurses in the midst of an influenza epidemic, to distinguish between these good women and really trained nurses?

Qualified by Government Certificate.

So far as the public are concerned, this is the chief thing we have to say regarding these unfortunate recommendations, namely, that if put into effect they will pour into the nursing field an annually increasing number of partially-trained women who, for all their lack of training, will be fully entitled to call themselves "qualified nurses." What more can anxious friends want, when they are telegraphing in all directions for a nurse? Surely one who is "qualified" as shown by Government certificate, who is certified as proficient by the superintendent nurse, and as "qualified to undertake the ordinary duties of a nurse" by the doctor attached to the "training school" ought to be all right; and thus it will happen on every side that people will, as the direct result of the recommendations of this committee, be led to accept as proper and efficient nurses women who would not be allowed to nurse a pauper except under supervision. It is easy to understand that the subdivision of the nursing staff of an infirmary into various grades, such as "probationers," "qualified nurses," "trained nurses," and "superintendent nurses" may be useful when each grade is allotted its own special duties beyond the limits of which it cannot pass; but when nurses are being scrambled for by an anxious but undiscerning public, the granting of misleading titles, such as those suggested in these recommendations, can but lead to confusion worse confounded.

The Injustice to Nurses at Large.

Let us now consider the injustice done by these proposals to the hardworking and deserving mass of women who, not merely at epidemic times when work is plentiful, but year in year out have to earn their living by the exercise of their profession and to put by out of their earnings some sort of a provision for old age. For many years back a steady movement has been in progress to raise the standard of training, to increase the efficiency, and as a result to elevate the status of the nurse. The time spent in being trained has been lengthened, the difficulty of the examinations has been increased, the width of knowledge demanded before a nurse is allowed to go out into the world to earn her living has been extended, and all this not in response to any demand from the public—who indeed so long as a nurse had a cap and a certificate were quite content—but solely because the conscience of the nursing profession rebelled at the idea of women undertaking responsible duties until they were thoroughly fitted to perform them. Hence the long laborious training which, at the cost of much trouble be it said, has gradually been rendered almost universal. And all this is to be undone merely that the guardians may be relieved of a difficulty in obtaining nurses! In the face of all that has been done to raise the standard of nursing, wherein lies the justice of swamping the market with these one-year-trained so-called "qualified nurses?" Doubtless it will serve the purposes of the Guardians very well to manufacture a large number of nurses year by year, and thus by increasing their multitude to lower their price. But how far it is right or even honest to employ the resources of a great public department to effect such an over-production of a class of labour of which the department itself is one of the chief employers, is another affair, especially when the scheme, if it is to effect its purpose, must result in turning out of their employment a large number of women who have spent their best years in properly fitting themselves for the honourable but laborious work of nursing the sick. Evidently the first object of the committee has been to find a way out in regard to workhouse nursing. All the rest has been to them a matter of indifference. But it is hard upon nurses as a class. No hesitation is made in advocating the employment of probationers on the ground that they are a cheap form of labour. "A probationer in return for her training and certificate is willing to give her services at a much less salary than similarly competent services could be obtained for under ordinary conditions. It is believed, therefore, that in many cases, by the employment of probationers, the sick in workhouses could be provided with a larger staff of nurses than the Guardians, from perhaps legitimate considerations of cost, could be induced to supply by the employment of none but ordinary paid nurses." That is plain enough.

The National Asset Question.

Again, we are told that it is evident that the nurses trained by the guardians become available in many cases for the nursing of sick other than the sick poor, and that "these nurses are a national asset, and the maintenance of the supply of them would seem to be essentially a national service to which Imperial funds should be called upon to contribute." We should imagine that engineers or any other class of "skilled workers" are also a "national asset," but we wonder what the engineers' union would say if Government were to take advantage of any public works which might be in hand to arrange for as much of the work

THE REPORT OF THE DEPARTMENTAL COMMITTEE ON WORKHOUSE NURSING—*Continued.*

as possible being done by apprentices, taken on for only a portion of the usual number of years, in order to increase this "national asset," and ensure that the next time Government had a similar job on hand there should be a glut in the market of that particular kind of labour which the Government was then likely to require! Yet that is just the idea in the minds of the committee. They hold that it is very desirable that the probationer class should be increased so as to permit of a surplus of "qualified nurses" (*i.e.*, the one-year-trained "nurses") being annually available, over and above the number required to fill the normal annual vacancies. "The probationer class should be regarded not only as the recruiting ground from which the normal annual demand for nurses can be supplied, but that it should also represent a kind of nursing reserve, from which in time of need an extra supply of nurses could be drawn." That is to say these nurses are to be manufactured wholesale and thrown out upon the public to live somehow—by competing with properly trained nurses of course—in order that they may be available when my Lords of the Poor Law may happen to require them. As a means of smoothing the wheels of poor-law administration, this is no doubt admirable; but to nurses trying to earn their living, it must appear quite the reverse—perhaps even the reverse of honest.

### The Isolation of Infectious Disease in a Private House.

### EXAMINATION QUESTIONS FOR NURSES.

The question for December was as follows:—What steps would you take to isolate a case of infectious disease in a private house to prevent the spread of infection?

### First Prize.

I should choose a room at the top of the house, if possible should retain the whole floor, for use of patient and attendants, otherwise should select the room furthest removed from rest of household.

This I should clear of all unnecessary furniture, remove carpet, curtains, and bed vallance.

A sheet kept damp, with 1 in 40 carbolic, should be hung inside and outside the door. This catches infected dust and serves as a reminder.

All utensils must be kept for patient's exclusive use. These should be cleansed by the nurse, so as to avoid being taken from the room, and the liability of getting mixed with others.

All bed and patient's clothing should be put at once into disinfectant.

All discharges from ears, nose, and throat should be burned, and motions and urine should be received in bed-pan containing 1 in 20 carbolic, 1 in 500 mercury, or 1 in 10 formaline, and more disinfectant added before emptying same. If motions are hard they must be broken up so that the disinfectant can well permeate.

The floor should be washed daily, and all furniture and ledges wiped with cloth damped with disinfectant. All remnants of food should be burned. A fire helps to ventilate the room. In scarlet fever, destroy any toys, books, or papers patient may have recently used.

Sponge skin daily, and anoint with antiseptic oil; this prevents fragments of skin getting into air. Use rags or absorbent wool for discharges from nose and throat, as these can be burned.

When convalescence is complete, patient should have two or three carbolic baths, and put on fresh clothes.

Room should be fumigated with sulphur and afterwards exposed to fresh air.

In small-pox the infection is contained mostly in secretions, excretions, skin, matter, and dried scabs. The skin should be bathed with disinfectant lotion. Scabs anointed with carbolised glycerine or vaseline, eyes, nose, and mouth cleansed with pieces of rag and at once burned. After patient is well should take antiseptic baths till free from infection. Disinfect room as for scarlet fever.

In diphtheria the infection is mostly contained in discharges from nose and throat. Avoid inhaling patient's breath, and take precautions when painting throat or cleansing tracheotomy tube to avoid receiving on lips or eyes any pieces of membrane that may be coughed up.

In enteric take precautions to disinfect motions, urine, sputum, and patient's skin, as the perspiration is a source of infection.

In all cases keep a bowl of disinfectant near bed for nurse's hands after attending to patient in any way.

The nurse should wear washable clothing and should avoid mixing with the rest of the household more than is necessary.—Daylight.

### Second Prize.

I should look out for a room or couple of rooms in the most secluded region of the house, on the top floor in preference, in which to isolate the patient, and, if possible, keep the whole floor for patient and myself only. Stripping the room and landing of all unnecessary furniture, curtains, carpets, or anything of a woolly material likely to harbour dust, except, of course, blankets necessary for warmth and comfort. I should daily scrub the patient's room with carbolic soap and should keep a sheet suspended outside the door, the end resting in an old foot-bath containing carbolic acid lotion, 1 in 20, with which to keep the sheet damp. All the patient's soiled linen I should place in a vessel containing either perchloride of mercury, 1 in 2,000, or carbolic, 1 in 20, and allow them to soak 24 hours before sending them to the laundry. With all excreta from patient I should put some carbolic, 1 in 20, before removing from room, and daily well flush with some strong disinfectant any closets or sinks I used. I should ask that our food be placed outside the door, and should destroy any food remnants by burning, keeping a fire in the room if the season permitted, and admitting plenty of fresh air through window. As soon as the doctor permitted I should give the patient a daily warm bath, and well rub the skin with carbolic or camphor oil, or vaseline, to prevent as far as possible the diffusion of desquamating particles. When the doctor pronounced desquamation finished, I should thoroughly well wash patient's head with perchloride of mercury, 1 in 2,000, and give a carbolic bath; then have an entirely fresh set of clothes for the patient to dress in, who would then be safe in meeting, but not in close contact, with other members of the household. In preparing the room for disinfecting, I should make it as air-tight as possible by covering up the window sashes, ventilators, doors, and fire-place, by pasting stiff paper over these openings, or filling them up with strips of rag stuffed in tightly. Get a piece of rope and nail up across the room to hang anything over belonging to patient, which has been in the room. The mattress and and blankets I should get stoved, if possible, by the sanitary authorities. Having the room ready, I should then place a sulphur candle, or candles, according to size of room, on an old fire shovel, resting on a bucket turned upside down; ignite the candle and close the room for 24 hours. In small-pox I should take the same precautions, and in addition should find out if the other inmates of the house had been recently vaccinated, and if not, should report to the doctor. Diphtheria and enteric being contagious only, it would not be necessary to have the carbolic sheet suspended over door, or carpets, etc., removed from any other than patient's bedroom, and a daily sponge in bed would have to be substituted for a daily bath.—"Ésperance."

### Mediocrity.

It has been very difficult to judge the papers this month with a view to prizes, because, unfortunately, none of them can be considered first-class, though many are very good. It is a serious fault that so few nurses have taken sufficient heed to the second part of the question, although a N.B.

drew special attention to it. Very few have differentiated adequately between scarlet fever, small-pox, enteric, and diphtheria. "Daylight" speaks most to the point on this subject, but I should like something to have been said concerning the importance of the nurse not eating or drinking without first cleansing her throat with Condy or some such disinfectant. "Esperance," the second prize winner, hardly speaks of diphtheria at all, but has practical ideas on the subject of moving the patient when the time comes.

Honourable Mention.

This is gained by three nurses, "Nell," "Santa Claus," and "Edith." "Santa Claus'" paper is very good on the whole and she would have been a prize winner but she said nothing concerning diphtheria.

Unsatisfactory Answers.

If there are a considerable number of answers above the average, there are, on the other hand, some deplorably bad ones, showing a total disregard of rules.

Question for January.

Pending the doctor's arrival, what precautions should you take in a case of fractured pelvis, and in what manner should you place him in the bed? The Examiner.

### Presentations.

West Ham Union Infirmary.—A presentation was recently made by the senior and leading officials of the West Ham Union Infirmary to Miss Clark, lady superintendent of nurses, on the termination of her services. Dr. Vallance, in making the presentation, consisting of a silver-backed toilet set and plaid rug, said he felt compelled to express his regret that Miss Clark had not seen her way clear to accept the position of matron at the new Infirmary, Whipps Cross. He also paid a warm tribute to the efficiency she displayed at all times in the discharge of her duties at West Ham Union Infirmary. The master of the Workhouse, Mr. Morgans, supplemented Dr. Vallance's remarks. He said that it had afforded him great pleasure to note the ready and most willing response made by the nurses, probationers, and others to subscribe towards the testimonial. It showed that Miss Clark had during her stay of four years with them succeeded in winning the good wishes of all with whom she was connected. Miss Clark becomingly acknowledged the presentation, and said that her duties had been considerably lightened by those with whom she worked.

### TRAVEL NOTES AND QUERIES.

Spain in March (Espagne).—If you cannot go beyond £7 or £8, it is useless to think of Madrid, which is £9 return without any extras. And how can you dream of Malaga, which would be very much more? Moreover, Malaga is not very picturesque. Madrid is bitterly cold in winter and spring, not particularly interesting except as to its art treasures, and certainly the journey is beyond your means. My advice is, go to San Jean de Luz, in beautiful mountain scenery, on the frontier and close to Fuentarabia, one of the most marvellonsly picturesque places I have ever seen. Second-class return to San Jean de Luz, £6 12s. or thereabouts. There is rather a cheaper way by taking the steamer to Bordeaux and on by rail, but in the end with porters, cabs, stewardess, etc. . . . my experience is that you do not gain sixpence, have a very disagreeable journey, and lose a great deal of time. If you do as I suggest, there is no reason at all why you should not visit San Sebastian as often as you like. Some travellers will tell you it is necessary to go first-class in Spain. It is perfect nonsense. I always go third (unless time is an object), and have always met with politeness. A carabinero usually bestows his company on you in the cause of law and order, which gives a sense of great security. Just this side of San Sebastian is an artistic gem called Passajes. You reach it from the station most primitively in a row boat. This place alone would supply you with sketching for a month. You could live at San Jean comfortably for the sum you mention. If this suits your ideas, write again and I will give you addresses, and some tips as to crossing the frontier, price of drives, etc.

### Appointments.

[No charge is made for announcements under this head, and we are always glad to receive, and publish, appointments. But it is essential that in all cases the school of training should be given.]

Camberwell Infirmary.—Miss Harriette Bell and Miss Ethel M. Whittle have been appointed sisters. Miss Bell was trained at Union Hospital, Bradford, and Miss Whittle at Chorlton Union Hospital.

Darlington Fever Hospital.—Miss Beatrice James has been appointed matron. She was trained at Liverpool Infirmary, and after holding various appointments at Manchester, Rotherham, and Birmingham became matron of Albert Infirmary, Winsford.

Derby Workhouse Infirmary.—Miss Agnes Mary Vincent has been appointed staff nurse. She was trained at Cranbrook Union Infirmary, and has since been assistant nurse at Eastville Workhouse, Bristol.

East Poorhouse Hospital, Dundee. — Miss Jessie Coutts has been appointed charge nurse. She was trained at Aberdeen City Hospital, and has since been engaged in private nursing in Aberdeen.

Inverness Northern Infirmary Convalescent Home. —Miss Elizabeth Massie has been appointed matron and housekeeper. She was trained at Cumberland Infirmary, and has since been night superintendent at the Northern Infirmary, Inverness.

Isolation Hospital, Sea Street, Herne Bay.—Mrs. A. Tellick has been appointed nurse in charge. She has been resident superintendent of Evesham Sanatorium, Stratford-on-Avon, and for 10 years matron of the Hove Hospital, Sussex.

McKelvie Isolation Hospital, Oban. — Miss Agnes Espie has been appointed nurse matron. She was trained at Paisley Infirmary and Edinburgh City Hospital. She has since been assistant matron at Ruchill City of Glasgow Fever Hospital.

New Isolation Hospital, Wimbledon. — Miss Flora Norton has been appointed sister. She was trained at St. Marylebone Infirmary, North Kensington, and temporarily acted as sister.

St. Alban's Hospital.—Miss Frances Alice Williams has been appointed staff nurse. She was trained at the Royal Infirmary, Wigan.

St. Helen's Sanatorium.—Miss Elizabeth Wright has been appointed sister. She was trained at the Borough Sanatorium, St. Helen's, for fever work, and at the Royal Infirmary, Sheffield.

The New Government Hospital, Pietersburg, Northern Transvaal, S.A.—Miss Bertha Sutcliffe has been appointed sister. She has lately been assistant matron at Burgher's Camp, Potchefstroom, Western Transvaal.

Victoria Cottage Hospital and Dispensary, Abergavenny.—Miss Winifred Jones has been appointed nurse-matron. She was trained at Taunton and Somerset Hospital, and has since been staff nurse at Newmarket Accident Hospital; charge nurse at Park Fever Hospital, Tottenham; and matron of Sidmouth Cottage Hospital. She holds the certificate of Apothecaries' Hall for dispensing.

West Ham and East London Hospital.—Miss Emily Mann has been appointed sister. She was trained at Durham County Hospital, and has since been staff nurse at Kendal Memorial Hospital.

### Echoes from the Outside World.

### The Prince of Wales's Fourth Son.

IF the new little prince who has come to join the family circle at York Cottage, Sandringham, had arrived five days later, he could have been truthfully called a Christmas gift. As it is, his advent on December 20th allows his father and his brothers and sisters to celebrate Christmas with easier minds and greater rejoicings, though nothing can make up to the children for the fact that their mother is not well enough to join in their fun. The Princess of Wales has always been devoted to her children, who repay her with much affection. The name of the new baby will be awaited with interest. The King's grandchildren are now seven in number. First in seniority are the two daughters of the Duke and Duchess of Fife, Princess Alexandra, born May 17th, 1891, and Princess Maud, April 3rd, 1893; then the Prince of Wales' family, Prince Edward, born June 23rd, 1894; Prince Albert, December 14th, 1895; Princess Victoria Alexandra, April 26th, 1897, and Prince Henry, born on March 31st, 1900.

### The Queen's Dinner.

ELABORATE arrangements are being made for the enjoyment of the Queen's guests on Saturday. The mënu is as follows: Hot roast turkey and sausages, hot sirloin of roast beef, boiled potatoes, brussels sprouts, Christmas pudding, mince-pie, grapes, bananas, oranges, apples, nuts, sweets, Christmas crackers, tea, coffee, cocoa, mineral waters. Each diner will be presented with a souvenir box of chocolate, and each child with a toy. A variety entertainment, organised by Mr. Moss of the Hippodrome, will be given on each of the three floors, and in all nearly 2,000 guests are expected. Special invitation cards have been issued, each bearing a portrait of the Queen. Professional waitresses—about 250—are to attend, as the organisers think it wiser to refuse the many offers of help from ladies who would like to have been of assistance. A special feature is to be made of the carrying round of a huge flaming plum-pudding on a silver salver. It is to be borne shoulder high by four chefs heading a procession of cooks, who will make a tour of the dining-rooms. The Queen has expressed a desire that two doctors and a number of nurses and ambulance men shall be in attendance on the occasion of the feast, lest their services should be required.

### Wireless Transatlantic Telegraphy.

ON Monday the interesting announcement was made that an official message had been transmitted by Signor Marconi's wireless telegraph from Lord Minto, Governor-General of Canada, to King Edward, and that Signor Marconi himself had transmitted a message by Lord Knollys for the King. A message from Marconi has also been sent to the King of Italy. The inventor has thus successfully established wireless telegraphic communication between Cape Breton and Cornwall. Lord Minto has already congratulated him upon his achievement, and other felicitations will not be slow to follow.

### The Humbert Frauds

After seven months' continuous search, not only throughout Europe, but in America, the Spanish police have succeeded in capturing the members of the Humbert family, who escaped from Paris last May. Madame Humbert, her sister and daughter, are now lodged in the women's prison in Madrid, whilst the two men have gone to the principal gaol. The prisoners protest that they are more sinned against than sinning, and that they "place their trust in French justice." It will be remembered that the originator of this tremendous fraud was the lady who is now Madame Humbert. She was the daughter of a Languedoc peasant-proprietor, and she stated that she was once able to assist a gentleman who was taken ill in the train. She afterwards discovered him to be an American millionaire, Henry Robert Crawford, who later, dying at Nice, in gratitude left her all his property, value £4,000,000. Then this extraordinary woman invented two nephews, whom she affirmed had turned up, contesting her claim and producing a new will. In order to defend her rights against these nephews, she borrowed large sums of money on the strength of her prospects, and for 22 years litigation has been going on between herself and the imaginary Crawfords. Ten judgments have been given, including two by the Supreme Court. During these 22 years Madame Humbert—who early in the suit married a rising young barrister, the son of the Minister of Justice—has raised loans to the extent of £2,000,000, including one from the Bank of France. Her method was to show coupons for bonds which she said were in a safe at her house, but in reality they were hired from various bondholders for the purpose. The fraud was only discovered by accident, the judge giving an order to allow a bridegroom to inspect the bonds in the safe, loans upon which comprised his wife's dowry. This exploded the bubble, and all connected with the Humberts at once fled the country.

### Christmas Plays.

AMONGST the attractions for the Christmas holidays "The Christian King" at the Adelphi and "The Water Babies" at the Garrick will probably be very popular. The hero of the former is naturally enough, as we have so recently been celebrating his millenary, King Alfred, and the events of his life are, as it were, arranged in a popular manner, with due regard to scenic display and also to the love interest, without which few plays are a success. In the first scene King Ethelred is borne on a litter into the courtyard, and with his last breath bequeathes to his brother, not only the Kingdom but also the gentle high-minded Elswitha. Alfred is determined to marry her, but Zebuda has come as a spy from the camp of Guthrum, King of the Northmen, in company with Cedroc, the Scandinavian, to bring about Alfred's destruction. However, instead of compassing her end she has learnt to love him. The play is occupied with various exciting episodes in which these two women figure largely, each contending for the King's love. Alfred is shown in the neatherd's hut, the scene of the legendary burning of the cakes. Ultimately Zebuda saves the lives of Alfred and Elswitha, dies herself, and Guthrum becomes a Christian and a friend of the King of England. Mr. Wilson Barrett is Alfred, and Miss Edyth Latimer Elswitha, whilst the character of Zebuda is well sustained, especially by Miss Lillah McCarthy.

"The Water Babies" is a charming dramatisation of Charles Kingsley's well-known book, produced by Mr. Arthur Bourchier, and adapted by Mr. Rutland Barrington. Few prettier Christmas plays have ever been seen; it is beautifully placed upon the stage, the acting is all that can be desired, the story is well brought out, and easy enough for even those children who have never read the book to grasp. The first scene shows poor Tom, as the chimney-sweep, frightening Ellie and her nurse by coming down the wrong chimney; the next he is in the Dames School at Vendale. The rest of the play takes place at the bottom of the sea, which is seen through gauze, where the prettiest of music is sung, and tiny feet dance the sweetest of dances. All children will delight in the new piece at the Garrick, and those who are no longer children will perhaps enjoy it even more.

### A Book and its Story.

PERSONAL REMINISCENCES OF AN IRISH WOMAN.*

Yesterday and to-day meet in lively contrast in Miss Harriet Corkran's very interesting memories of persons and events with whom, in the course of a varied life, she has been brought into immediate contact. Her childhood was passed in Paris, and the notable persons of whom she writes at that period were friends of her parents. Being a typical little Irish woman, vivacious and imaginative, keenly observant, and with a turn for caricature, she began early to make mental pictures also of her neighbours, which survive in the collection of anecdotes recently published as "My Celebrities and I." Many of the celebrities have long since joined the majority, but her recollections revive interest in those of whom one would imagine the last word had been said. For instance, of the Brownings, Tennyson, Thackeray, Rossetti, and others, she has lively personal memories which are very much to the point, and throw light on some much misunderstood persons judged solely by their books. Thackeray so antipathetic to some minds, as an acrid cynic, appears here as a kindly, and most genial man, who entered into the joys and sorrows of a child's life with the sympathy of a contemporary. Miss Corkran recalls at this time the impression which Thackeray's delightful personality made on her as being more deep and lasting than that of Robert Browning, whom she knew more or less intimately all her life. She writes: "No one made such a vivid impression on my childish imagination as Mr. Thackeray. He is the central figure which stands out more or less from the blurred surroundings. He had a formidable appearance, being over six feet and broad in proportion. I distinctly recall the big head with the silvery hair, the rosy face, the spectacles, and the sunny sweet smile which illuminated his face and made it beautiful. I had heard of the famous 'Vanity Fair,' and wondered why a man so celebrated should care to talk to us little children, and even play games with us in such a kindly, simple way." One afternoon when the little Harriet was passing with her father a famous English pastrycook's shop in La Rue du Luxembourg, she cast a longing glance at the cakes in the window. Her father, who was an absent-minded man, took no notice of this until suddenly aroused by a voice which seemed to her to be "something like an angel's from heaven" saying, "Oh, give her a tart!" "Hullo, Thackeray! did not know you were in Paris," replied Mr. Corkran. When Thackeray had conducted her solemnly into the shop, giving her the command to eat as many tarts as she wished, she exclaimed, "How nice to be always hungry and have as many tarts as one can eat." Mr. Thackeray's spectacles twinkled with fun. He glanced at the plum cakes in the window, "Dear old plum cakes!" he exclaimed, "how they remind me of my school-days," and he took off his hat and bowed comically to them. Another side of the great satirist's character is seen at the same moment, when, suddenly leaving the little girl to devour her good things, he went towards a very poor, delicate working woman with a baby in her arms, who was leaning for support against a tree, and after speaking to her, slipped a five-franc piece into her pocket. These are only a few of the Thackeray stories which show the warm heart and willing hand ever ready to do a kind action. She adds, "With children he was always delightful; it was only to older people that he could be satirical, cold, and cynical."

There are many references to the Browning family. At that time the poet's father was living in Paris in charge of his interesting and devoted daughter, Sarianna, who, still alive, "is a picturesque old lady with snowy hair over which she wears a soft white lace mantilla; her talk is full of zest and point, for she expresses herself remarkably. There is something of the old Trojan about her; she never complains or gives in to ill-health, and is always ready to sacrifice herself for those she cares for. . . . Her way of relating an anecdote is unique. She has also that rare quality of sound common sense flavoured now and then with flashes of inconsistency which makes her a truly delightful companion." Miss Corkran was still a child when she first met Robert and Elizabeth Barrett Browning. Having formed her ideas of the orthodox appearance of a poet from the classic models of Dante and Tasso, it was a shock to preconceived visions when, being told by her mother that two poets were coming to see her one afternoon, upon the door being opened by their French servant, with the announcement, "Monsieur et Madame Brunig," she saw to her great disappointment two persons as far removed outwardly in appearance from the poets of her dreams as the dress of the day could make them. "A frail little lady entered, in a simple grey dress and straw bonnet, followed by a cheerful gentleman in a brown overcoat!" "Could this conventional pair be the two great poets?" she asks in surprise. Later, after a visit to them, her disillusionment was complete as to the ethereal nature of the poet at least. "It gave me a shock to see Mr. Browning eat with avidity so much bread and butter and big slices of plum cake. He never said a word that suggested a poetical thought, and his voice was loud and cheerful, his thick hair well brushed. "Altogether," she adds, "he looked, in my opinion, like a a prosperous man of business." From his mother the poet inherited his love of music, for she was a gifted musician, and his old retiring, shy father was, to Miss Corkran, far more like a man of genius, with his quaint air of simplicity and unworldliness, than his celebrated son Robert. Later reminiscences are of him in Warwick Crescent. One afternoon, when calling upon Miss Browning, she saw from the window of the back drawing-room the curious spectacle of Robert Browning carrying a goose in his arms. "Ah! our pet has been ill," said Miss Browning, "so Robert is looking after it." Miss Corkran remarked that it was a singular kind of pet. "It is so clever and affectionate," answered Mr. Browning, "and follows me about just like a dog, does he not Sarianna"? appealing to his sister. Dante Rosetti and Swinburne formed an arresting contrast when seen together one evening in "dear old Bohemia." "Rosetti, dark, mysterious, magnetic; Swinburne, fair, bright-eyed, gesticulating wildly as if he had quicksilver in his veins. Rosetti had rather a massive figure; Swinburne was slight and small, his big head covered with a forest of towsing hair, which he tossed about wildly." The authoress closes her book with the words: "There is one merit in this record—*i.e.*, sincerity. I have invented nothing. It is all true." Truth, as presented by Miss Corkran, is more interesting than any fiction.

* "My Celebrities and I." By H. Corkran. (Publishers: Hutchinsons. 1 Vol. Price 7s. 6d.)

### To Nurses.

We invite contributions from any of our readers, and shall be glad to pay for "Notes on News from the Nursing World," or for articles describing nursing experiences, or dealing with any nursing question from an original point of view. The minimum payment for contributions is 5s., but we welcome interesting contributions of a column, or a page, in length. It may be added that notices of appointments, entertainments, presentations, and deaths are not paid for, but that we are always glad to receive them. All rejected manuscripts are returned in due course, and all payments for manuscripts used are made as early as possible after the beginning of each quarter.

### For Reading to the Sick.

"LOVE CAME DOWN AT CHRISTMAS."

LOVE came down at Christmas,
Love all lovely, Love Divine,
Love was born at Christmas,
Star and Angels gave the sign.

Worship we the Godhead,
Love Incarnate, Love Divine,
Worship we our Jesus—
But wherewith for sacred sign?

Love shall be our token,
Love be yours and love be mine,
Love to God and all men,
Love the universal sign.

*C. Rosetti.*

Man longs for God. And it is the blessedness of revelation that it brings before us, in all the vicissitudes of history, in all the chronicles of patriarchal life, in all the solemn tones of teaching and sorrow which come from the Prophets, in the history of the Church, above all, in the life of our Master, the great rebuking, consoling thought of One Who changes not, and never fails us—the thought of God.

God loves us, not because we are what we are, but because He is what He is. To drink in the depth of that revelation will need an eternity; but surely, surely, as the thirsty land drinks the rain in summer, as the flower drinks the sunshine in the morning of spring, as you and I drink delight from the face, from the voice, of one we love, as sweet music carries the soul into the undescribed dominions of unfettered fancy and untroubled thought, as the light of stars is tender when we think beneath it, as the summer sea, as the withering grasses and the bending corn give a sense of peace, and whisper stories from a distant land, coming in the quiet evening, flitting by us in the falling light; so, so to the heart of the sorrow-laden and weary, so to the misunderstood and misinterpreted, so to the sick and mournful, so to the sin-laden and sad, comes with a power of more than music the thought which is the most blessed in all the records of revelation—"God loves *me*;" for "God is love."—*Knox Little.*

Fling wide the portals of your heart,
Make it a temple set apart
From earthly use for Heaven's employ,
Adorned with prayer, and love, and joy.
So shall your Sovereign enter in,
And new and nobler life begin.

*Anon.*

Lord, give me blessed fear,
And much more blessed love,
That fearing I may love Thee here
And be Thy harmless dove:

Until Thou cast out fear,
Until Thou perfect love,
Until Thou end mine exile here
And fetch Thee home thy dove.

*C. Rosetti.*

### Notes and Queries.

**The Editor is always willing to answer in this column, without any fee, all reasonable questions, as soon as possible.**

**But the following rules must be carefully observed:—**

**1. Every communication must be accompanied by the name and address of the writer.**

**2. The question must always bear upon nursing, directly or indirectly.**

**If an answer is required by letter a fee of half-a-crown must be enclosed with the note containing the inquiry, and we cannot undertake to forward letters addressed to correspondents making inquiries. It is therefore requested that our readers will not enclose either a stamp or a stamped envelope.**

*Plague Duty.*

(97) Will you kindly tell me to whom to apply for an appointment for plague duty in India?—*A. P.*

The Secretary, the India Office, St. James's Park, S.W.

*Johannesburg.*

(98) Will you please inform me the address of the new Co-operative Association recently started in Johannesburg?—*M. D.*

The Superintendent Nurse, the Co-operative Residential Club, Johannesburg. But there are no vacancies at present.

*Tallerman.*

(99) Can you tell me anything concerning the "Tallerman" treatment for rheumatoid arthritis, and where I could apply for particulars?—*M. B. D.*

Apply to the Tallerman Institute, 50 Welbeck Street, Cavendish Square, W.

*L.O.S.*

(100) I am working as district nurse in this place, and as I often have to take maternity cases without a doctor I should like to get the L.O.S. Will you tell me the quickest and best way to do so? I do not wish to give up my post here if possible, at the same time I should like to learn the work thoroughly, and I cannot afford to spend very much.—*L. E.*

Write to the Secretary of the London Obstetrical Society, 20 Hanover Square, London, W., and ask for the syllabus of the examination required; then consult your local medical man, and ask him to prepare you for it. You might also write to the Secretary of the Midwives' Institute, 12 Buckingham Street, Strand, W.C., as she would be able to give you the best advice under your circumstances.

*Maternity Work.*

(101) Can you kindly give me the name of a good agency or association through which I could obtain private monthly cases? I am a fully-trained midwife and monthly nurse, but I have no general training.—*B.*

General training is now an essential qualification for employment by all the best nurses' associations. Try and work up a connection through private introductions, and apply to any of the private associations advertising in our columns which might be suitable.

*Nævus.*

(102) Can you tell me of any good hospital in the neighbourhood of Lancaster, Manchester or Liverpool where a little boy of seven could be properly treated for a bad nævus just below the right eye?—*M. C. R.*

Either of the great general hospitals in Manchester or Liverpool

*Short Training.*

(103) *M. L.* would be glad to know if she can be received into a hospital for one year's general training without having to pay a premium.

None of the larger hospitals would receive *M. L.* on such terms Her only chance is to apply to some of the small cottage hospitals.

**Standard Nursing Manuals.**

"The Nursing Profession: How and Where to Train." 2s. net; post free 2s. 4d.

"A Handbook for Nurses." (Illustrated). 5s.

"Nursing: Its Theory and Practice." (New Edition.) 3s. 6d.

"Nursing in Diseases of Throat, Nose, and Ear." 2s. 6d.

"Surgical Ward Work and Nursing." (Revised Edition.) 3s. 6d. net; post free, 3s. 10d.

"Art of Massage." (Second Edition.) 6s.

"Elementary Physiology for Nurses." 2s.

"Elementary Anatomy and Surgery for Nurses." 2s. 6d.

"Practical Handbook of Midwifery." 6s.

"Mental Nursing." 1s.

"Art of Feeding the Invalid." 1s. 6d.

All these are published by the SCIENTIFIC PRESS, Ltd., and may be obtained through any bookseller or direct from the publisher, 28 and 29 Southampton Street, London, W.C.

### Travel Notes.

**By Our Travelling Correspondent.**

### CXV.—SUNSHINE AND CLOUD IN FLORENCE.

Beware of the cold in Florence, have plenty of thoroughly good wraps, woollen underclothes always, light woollen dresses, and carry a cape over your arm on warmer days to put on when you enter shady streets (which strike like a vault after the warm sun), and also for use in the churches. Attention to these apparently trifling matters, insure keeping "fit," and for those who are not robust they are of the first importance. It can be bitterly cold in Florence when the snow-laden wind sweeps down from the mountains, but it is clear and dry and the large amount of sun makes it possible to be in the air nearly all day, a privilege so dear to the anæmic and those suffering from depression.

### How One Gets Milk for Tea.

If you are catering for yourselves, or even in an hotel, it is well to arrange tea privately as it is an extra charge. Many of the noble Florentine families, somewhat crippled in their resources—a state by no means unknown in our own country—let their magnificent palaces in flats, reserving to themselves the cellars, etc., where they thriftily install a trusted servant who sells for them the produce of their country dairy. In these quaint subterannean shops we plebeian foreigners can supply our base needs. Delicious butter is to be had, and excellent milk. The noble merchant supplies you with a small pear-shaped bottle in which, according to size, you carry off your milk. N.B.—You can have as little as a halfpenny-worth. The next day you bring the bottle back—you are not even asked to wash it!—and you take a fresh one. It is very satisfactory to know that one's milk and butter has come straight from its aristocratic home, and has not passed through the deteriorating hands of middlemen. The Italian noble is a simple creature, who does not imagine his dignity will be in the least endangered by your knowing his poverty and seeing how he augments his illustrious revenues; and he is quite right. There are none of those ignoble shifts of false names—so-called companies, etc.—which attempt in England to hide from the public gaze the dire disgrace of combined poverty and trade.

### Alas! the Mercato Vecchio!

The interest of Florence has not suffered to anything like the same extent as Rome from senseless demolitions and still more atrocious creations; it is very much less altered, but there are some interesting landmarks gone which we must ever regret. Such is the Mercato Vecchio with the Ghetto attached. It was, twenty years since, a mine of artistic beauty and, one must add, terrible dirt; now all that is left to mark its site is a detestable new piazza and on one of the houses the celebrated "Devil" of John of Bologna.

Howells gives us a description of the picturesque squalor of the place not long before its demolition:—

> I seem to stand in the midst of the old square with its mouldering colonnade on one side, and on the other its low, irregular roofs, their brown tiles thinly tinted with a growth of spindling grass and weeds. In front of me a vast white old palace springs seven stories into the sunshine, disreputably shabby from basement to attic, but beautiful with the rags of a plebeian wash-day caught across it from balcony to balcony as if it had fancied trying to hide its forlornness in them. Around me are peasants and donkey-carts and Florentines of all ages and sizes; my ears are filled with the sharp din of an Italian crowd, and my nose with the smell of immemorial, innumerable market days and the rank, cutting savour of frying fish and cakes from a score of neighbouring cook-shops. . . . Through an archway in the street behind me, not far from an admirably tumble-down shop full of bric-a-bric of low degree, all huddled—old bureaus and bedsteads, crockery, classic lamps, assorted saints, shovels, flat-irons, and big-eyed Madonnas—under a sagging peat roof, I enter a large court like Piazza Donati. . . .

It was in this quarter that the powerful Medici had their first houses, from here that they became money-lenders to the world, and on account of their origin from a physician used three pills as their family insignia. As all the world knows, the three gilded balls are now the recognised sign of a pawnbroker.

### The Brothers of the Miserecordia.

In watching a funeral or other work of mercy conducted by this singular confraternity, one is projected backwards many hundreds of years. So unusual a band seems strangely at variance with our matter-of-fact utilitarian manner of conducting life in the twentieth century. Originally founded early in the thirteenth century, the Order gave itself entirely to works of mercy, nursing the sick, succouring the afflicted, and burying the dead. Its singularity consists in the fact that the Brothers are men of all ranks, remain in the world and are or can be if they desire it, unknown to each other. It is popularly supposed that many of those who belong to it and who religiously carry out all the obligations of hasty attendance at accidents and at the burying of the dead, are those who feel a deep repentance for some wrong-doing, and would fain make some expiation by these works of mercy.

At the ringing of the bell any member, even if at a wedding or a ball silently steals away to the Oratory of the Miserecordia, which is close to the Duomo, dons his habit and mask and assists in whatever is needed. The dress consists of a perfectly concealing black robe from neck to heels, and a hood, also of black, falling in a long point down to the breast, having no opening but two slits for the eyes. Funerals usually take place towards evening and the effect is most weird and sinister; some twelve brothers attend, four carry the coffin, four walk in front with flaming torches, and four behind. At times they suddenly reverse the torches and raise them again with a flash, whilst they pass you unrecognised, for who can identify a man of whom nothing is seen but a pair of glittering eyes through the mask. I must say such a funeral is a very impressive sight, and I should have thought it would have been most terrifying to little children; but no, they see it from their earliest infancy and "familiarity breeds contempt."

The rapidity with which these men appear on the scene of an accident is surprising. I was watching something from my window, which afterwards proved to be a suicide (though at the time I could not see what was going on) from the banks of the Arno. I heard a bell ringing faintly, and it could not have been more than five minutes before three of these brothers of mercy were on the scene doing everything possible for the unfortunate victim of despair.

### "The Hospital" Convalescent Fund.

The Honorary Secretary begs to acknowledge with thanks the receipt of 5s. from Nurse Phœbe Simmons, and also her kind promise to become a regular subscriber to this Fund.

